# Cuproptosis-related lncRNA scoring system to predict the clinical outcome and immune landscape in pancreatic adenocarcinoma

**DOI:** 10.1038/s41598-023-47223-4

**Published:** 2023-11-27

**Authors:** Yi Huang, Ping Gong, Li Su, Mei Zhang

**Affiliations:** 1 Oncology Department of Integrated Traditional Chinese and Western Medicine, The First Affiliated Hospital of Anhui Medical University, Hefei, China; 2 Internal Medicine Department of Oncology, Anhui Wannan Rehabilitation Hospital (The Fifth People’s Hospital of Wuhu), Wuhu, China

**Keywords:** Cancer, Genetics, Immunology, Molecular biology, Diseases, Gastroenterology, Oncology

## Abstract

Cuproptosis is a recently discovered novel programmed cell death pathway that differs from traditional programmed cell death and has an important role in cancer and immune regulation. Long noncoding RNA (lncRNA) is considered new potential prognostic biomarkers in pancreatic adenocarcinoma (PAAD). However, the prognostic role and immune landscape of cuproptosis-related lncRNA in PAAD remain unclear. The transcriptome and clinical data of PAAD were obtained from The Cancer Genome Atlas (TCGA) database. Cuproptosis-related lncRNA was identified using Pearson correlation analysis. The optimal lncRNA was screened by Cox and the Least Absolute Shrinkage and Selection Operator (LASSO) regression mode, and for the construction of risk scoring system. PAAD patients were divided into high- and low-risk groups according to the risk score. Clinicopathological parameter correlation analysis, univariate and multivariate Cox regression, time-dependent receiver operating characteristic (ROC) curves, and nomogram were performed to evaluate the model. Gene Ontology (GO) and Kyoto Encyclopedia of Genes and Genomes (KEGG) analyses were used to explore differences in biological function between different risk groups. Single-sample gene set enrichment analysis (ssGSEA) and Estimation of STromal and Immune cells in MAlignant Tumor tissues using Expression data (ESTIMATE) algorithm were used to analyze the differences in tumor immune microenvironment (TIME) in different risk groups of PAAD. Additionally, the Tumor Immune Dysfunction and Exclusion (TIDE) algorithm was used to predict immunotherapy response and identify potential immune beneficiaries. Immune checkpoints and tumor mutation burden (TMB) were also systematically analyzed. Finally, drug sensitivity analysis was used to explore the reactivity of different drugs in high- and low-risk groups to provide a reference for the selection of precise therapeutic drugs. Six cuproptosis-related lncRNAs (AL117335.1, AC044849.1, AL358944.1, ZNF236-DT, Z97832.2, and CASC8) were used to construct risk model. Survival analysis showed that overall survival and progression-free survival in the low-risk group were better than those in the high-risk group, and it is suitable for PAAD patients with different clinical characteristics. Univariate and multifactorial Cox regression analysis showed that risk score was an independent prognostic factor in PAAD patients. ROC analysis showed that the AUC values of the risk score in 1 year, 3 years and 5 years were 0.707,0.762 and 0.880, respectively. Nomogram showed that the total points of PAAD patients at 1 year, 3 years, and 5 years were 0.914,0.648, and 0.543. GO and KEGG analyses indicated that the differential genes in the high- and low-risk groups were associated with tumor proliferation and metastasis and immune regulatory pathway. Immune correlation analysis showed that the amount of pro-inflammatory cells, including CD8^+^ T cells, was significantly higher in the low-risk group than in the high-risk group, and the expression of immune checkpoint genes, including *PD-1* and *CTLA-4*, was increased in the low-risk group. TIDE analysis suggests that patients in the low-risk group may benefit from immunotherapy. Finally, there was significant variability in multiple chemotherapeutic and targeted drugs across the risk groups, which informs our clinical drug selection. Our cuproptosis-related lncRNA scoring system (CRLss) could predict the clinical outcome and immune landscape of PAAD patients, identify the potential beneficiaries of immunotherapy, and provide a reference for precise therapeutic drug selection.

## Introduction

With the discovery of immune checkpoint inhibitors, immunotherapy is considered to have promising applications^1^. However, clinical studies have shown that immunotherapy has no effect on pancreatic cancer, known as the “immune desert” tumor^2^. The hypoxic tumor microenvironment of pancreatic cancer leads to an adverse inflammatory microenvironment and low immunogenicity^3,4^, hindering the extent of local infiltration by pro-inflammatory cells, such as natural killer (NK) cells, CD4^+^ T cells, CD8^+^ T cells, M1 macrophages, and dendritic cells^5^. This is the reason why most immunotherapy treatments for pancreatic cancer fail. Nevertheless, there are still patients who are beneficiaries of immunotherapy for pancreatic cancer^6^. Therefore, populations that would potentially benefit from immunotherapy are identified based on the clinical characteristics and tumor immune microenvironment (TIME), which identification is important to improve the survival prognosis of pancreatic cancer patients.

Although the content of copper ions in the human body is very low, it is involved in many biological activities, such as anti-oxidation, cellular metabolism, and mitochondrial respiration^7^. The role of copper ions in cancer and immunomodulation was discovered in the 1970s^8^. Previous studies have shown that copper can contribute to cancer progression by promoting angiogenesis and mediating the BRAF signaling pathway. The use of copper chelators to reduce intracellular copper uptake causes a decrease in mitochondrial reactive oxygen species levels in pancreatic cancer cells, causing the cells to become dormant^9^. The reduction in copper levels also significantly increases the number of CD8^+^ T lymphocyte and NK cells, promotes ubiquitin-mediated PD-L1 degradation, and inhibits tumor growth^10^. This evidence demonstrates the important role of copper in immunotherapy. Tsvetko et al., in March 2022, first observed that excessive copper ions could cause cancer cell death. The mechanism may be that an excess of copper ions impairs mitochondrial respiratory function, leading to the aggregation of lipidated and destabilized proteins of Fe–S cluster proteins, which, in turn, results in proteotoxic stress and, ultimately, cell death^11^. This process of cell death was named cuproptosis. Abnormally high serum concentrations of copper have been reported in pancreatic cancer, suggesting that factors associated with copper death might be a potential biomarker^12^. However, the regulatory role of cuproptosis in pancreatic cancer remains unclear.

Long noncoding RNA (lncRNA), which is mainly transcribed by RNA polymerase II, is a class of RNA consisting of ≥ 200 units of nucleotide^13^. Although it does not directly encode a protein, it is closely associated with chemotherapy resistance, immune escape, angiogenesis, and regulation of the TIME^14–16^. lncRNA is differentially expressed in pancreatic cancer tissues and normal tissues^17^, influence the proliferation and migration of pancreatic cancer, have a significant correlation with survival^18,19^, and are considered a new potential prognostic biomarker. In addition, lncRNAs have been shown to inhibit the progression of pancreatic cancer and reverse drug resistance by regulating programmed cell death such as autophagy and ferroptosis^20, 21^. However, the regulatory role of lncRNAs in curoptosis remains unclear. Previous researchs have confirmed that curoptosis-related lncRNAs and their model features have been shown to accurately predict the clinical outcome and immune landscape of hepatocellular carcinoma^22^, lung adenocarcinoma^23^ and endometrial cancer^24^, and can predict the response to immunotherapy through immune checkpoint genes. Therefore, we further explored the role of curoptosis-related lncRNA related scoring system in predicting tumor immune microenvironment and immunotherapy response in pancreatic cancer.

In this study, we constructed the cuproptosis-related lncRNA scoring system (CRLss) to stratify pancreatic adenocarcinoma (PAAD) patients by risk scores. Furthermore, we systematically explored the predictive value of CRLss for the prognosis, TIME, and immunotherapy response in PAAD patients to provide a basis for individualized treatment planning.

## Materials and methods

### Data collection

By searching the Cancer Genome Atlas (TCGA) database (http://cancergenome.nih.gov), transcriptome expression data (including 4 normal and 179 tumor samples), clinical information data (n = 185, including survival, age, grade, and TNM stage), and gene mutation data (n = 169) were extracted from the TGCA-PAAD cohort. Progression-free survival (PFS) data were retrieved from the Xena Explore (https://xenabrowser.net/) database at the University of California, Santa Cruz. Ten cuproptosis-related genes (CRGs) selected for this study were derived from previously reported studies^11^. A protein–protein interaction (PPI) network for those 10 CRGs was constructed using the String (https://cn.string-db.org/) database. Strawberry perl (version 5.32.1.1) was used to integrate matrix files for transcriptome (including mRNA and lncRNA) expression and mutation data. Autophagy related genes from MSigDB database (http://www.gsea-msigdb.org/gsea/msigdb/) of the Human Gene Set: GOBP_REGULATION_OF_AUTOPHAGY.

### Identification of cuproptosis-related lncRNA

First, we extracted the expression of ten CRGs in the TCGA-PAAD cohort. The correlation coefficient between CRGs and lncRNA was calculated by Pearson correlation analysis. We used | correlation coefficient | of > 0.3 and a p-value of < 0.001 as the threshold to obtain cuproptosis-related lncRNA. Visualization was performed with Cytoscape (version 3.8.0) software. Additionally, we integrated expression and survival data of cuproptosis-related lncRNA in the TGCA-PAAD cohort for subsequent analyses. The above-mentioned analyses were performed by using R software “limma,” “dplyr,” “ggalluvial,” and “ggplot2” packages.

### Construction of CRLss

First, we divided the TCGA-PAAD integrated datasets randomly into a training cohort and a test cohort in a 1:1 ratio. Next, we screened for survival-associated lncRNA by performing Cox survival analysis on the training cohort. Then Least Absolute Shrinkage and Selection Operator (LASSO) regression analysis was performed on training cohort with survival-related lncRNA. Specifically, we constructed a penalty function to get a more refined model through cross-validation to find the minimum λ value mapping of lncRNAs^25^, and these lncRNAs were used to build CRLss. The risk score was calculated as follows:

$${\text{score = expression of a lncRNA [1] }} \times {\text{corresponding coefficient of a lncRNA [1] + expression of a lncRNA [2]}} \times {\text{corresponding coefficient of a lncRNA [2] + }} \cdots {\text{ + expression of lncRNA [n]}} \times {\text{corresponding coefficient of a lncRNA [n]}}{.}$$

In the equation, the expression (i) and corresponding coefficient (i) represent the expression and Cox regression coefficient in CRLss. According to the median risk score in the training cohort, the training and testing cohorts were divided into high-risk and low-risk groups, and the testing cohort was used as a validation set to evaluate the predictive performance of the CRLss.

### Validation of the CRLss 

We first assessed the clinical baseline variability of the entire cohort, training cohort, and testing cohort to validate the predictive performance of the CRLss. With the R software “limma,” “scatterplot 3d” package, allGene, cuproptosis-related gene, cuproptosis-related lncRNA, and risk lncRNAs were used as the main characteristics to perform principal component analysis (PCA) of CRLss. Then, K-M survival analysis (including OS and PFS), risk curves, and risk heat maps for different cohorts were used to further verify the predictive performance of CRLss. Given the close association between cuproptosis and autophagy, we also explored the correlation between risk lncrnas and autophagy-related genes using Pearson correlation analysis ( | correlation coefficient |> 0.5 and *P* < 0.001 ).

In addition, we also used the R software “survival” and “survminer” to identify the survival correlation of different clinical characteristics, including age, gender, pathological stage, and grading, in the high- and low risk-groups of CRLss, which was used to evaluate whether the constructed CRLss was applicable to different clinical groups of PAAD patients. Univariate and multifactorial COX regression analyses were used to assess whether risk score and other clinical characteristics were independent prognostic factors. We calculated their concordance index (C-index) through the R package “dplyr,” “survival,” “rms,” and “pec,” which was used to evaluate their predictive ability in the model.

### Identification of clinical value of CRLss in PAAD

With the help of the R package “TimeROC,” “Survival,” and “Survminer,” the 1-, 3-, and 5-year ROC survival curves in the CRLss were plotted. Area under the curve (AUC) were used to evaluate the clinical prognostic value of the CRLss. Clinicopathological parameters were also stratified as subgroups for analysis. Additionally, based on the results of uni- and multi-factor Cox analysis, logistic model and Cox proportional risk model, we constructed a nomogram consisting of risk score, clinical features, and survival prognosis to predict 1-, 3-, and 5-year OS in PAAD patients. A calibration curve based on the Hosmer–Lemeshow goodness of fit test was used to assess the clinical credibility of the nomogram.

### Enrichment analysis of differential genes

With | log2 fold change (FC) | of > 1 and *p* of < 0.05 as the threshold, differentially expressed genes (DEGs) in the high- and low-risk groups of CRLss were identified. Gene Ontology (GO) and Kyoto Encyclopedia of Genes and Genomes (KEGG)^26^ enrichment analyses were performed to explore the biological functions of DEGs.

### Immunocorrelation analysis of CRLss

TIME is closely related to the occurrence and progression of cancer. Immune cell infiltration and stromal cell metabolism in the tumor microenvironment have a profound influence on the TIME^27^. The ESTIMATE algorithm was used to calculate the abundance of immune cells and stromal cells in tumor tissue, as well as the purity of tumor tissue^28^. Next, the immune function scores of the tumor samples were calculated using ssGSEA^29^, and the differences between tumor microenvironment and immune function scores in different risk groups of CRLss were analyzed using the R software “reshape2” and “ggpubr” packages.

In addition, the degree of immune cell infiltration is one of the indicators to predict the immunotherapy response, which is closely related to the prognosis and survival of pancreatic cancer^30^. CIBERSORT is the most frequently cited tool for estimating immune cell infiltration^31^. We used the R language “CIBERSORT” package for deconvolution analysis of the gene expression matrix of immune-related cell subtypes. We set the perm value to 1000 to ensure the accuracy of the results. Then, the infiltration abundance of different immune cells in tumor samples was calculated, and the correlation between CRLss risk score and immune cells was analyzed.

### Immunotherapy response predictions for CRLss

Immunotherapy response refers to immune checkpoint inhibitors binding with corresponding immune checkpoint genes on tumor cells to activate the immune recognition and immune response of T cells to tumor cells to kill tumor cells^32^. Based on this, we explored the differential expression levels of immune checkpoint genes in high- and low-risk groups of the CRLss.

Tumor Immune Dysfunction and Exclusion (TIDE) is a newly developed computational method for predicting an immunotherapy response^33^. We obtained the scores in the TCGA-PAAD samples from the TIDE (http://tide.dfci.harvard.edu/) database and analyze the variability of scores in different risk groups of the CRLss for predicting the immunotherapy response in the model.

Tumor mutation burden (TMB) is defined as the total number of somatic gene coding errors, base substitutions, and insertion or deletion errors detected per million bases. There is growing evidence that TMB expression levels correlate with the efficacy and prognosis of PD-1/PD-L1 inhibitors in selected tumors^34,35^. Therefore, we explored the difference in TMB expression in CRLss high- and low-risk groups. Next, the R “survival” and “survminer” packages were used to obtain the optimal cutoff of TMB, which was used to plot the K-M survival curves of TMB in different risk groups of the CRLss.

### Drug sensitivity analysis

R “pRRophetic,” “ggpubr,” and “limma” packages were used to obtain the half-maximal concentration (IC50) of the drug in the high- and low-risk groups to identify the difference in drug sensitivity of different risk groups of the CRLss. Then we screened clinically commonly used drugs for presentation by drawing box plots.

### Statistical analysis

Strawberry version of perl (version 5.32.1.1), R software (version 4.1.2), and related packages were used for statistical analysis of data and graphing. Cytoscape (version 3.8.0) was used to visually demonstrate the correlation between 10 cuproptosis-related genes and 34 cuproptosis-related lncRNAs. Wilcoxon’s and Kruskal–Wallis were used to compare differences between groups using Pearson’s correlation coefficient to assess correlations between variables, and Kaplan–Meier and Cox regression models were used for survival correlation analysis. All statistical P-values were bilateral, and a *P* of < 0.05 was considered statistically significant without special note.

## Results

### Screening of cuproptosis-related lncRNAs in PAAD

**Figure 1:**
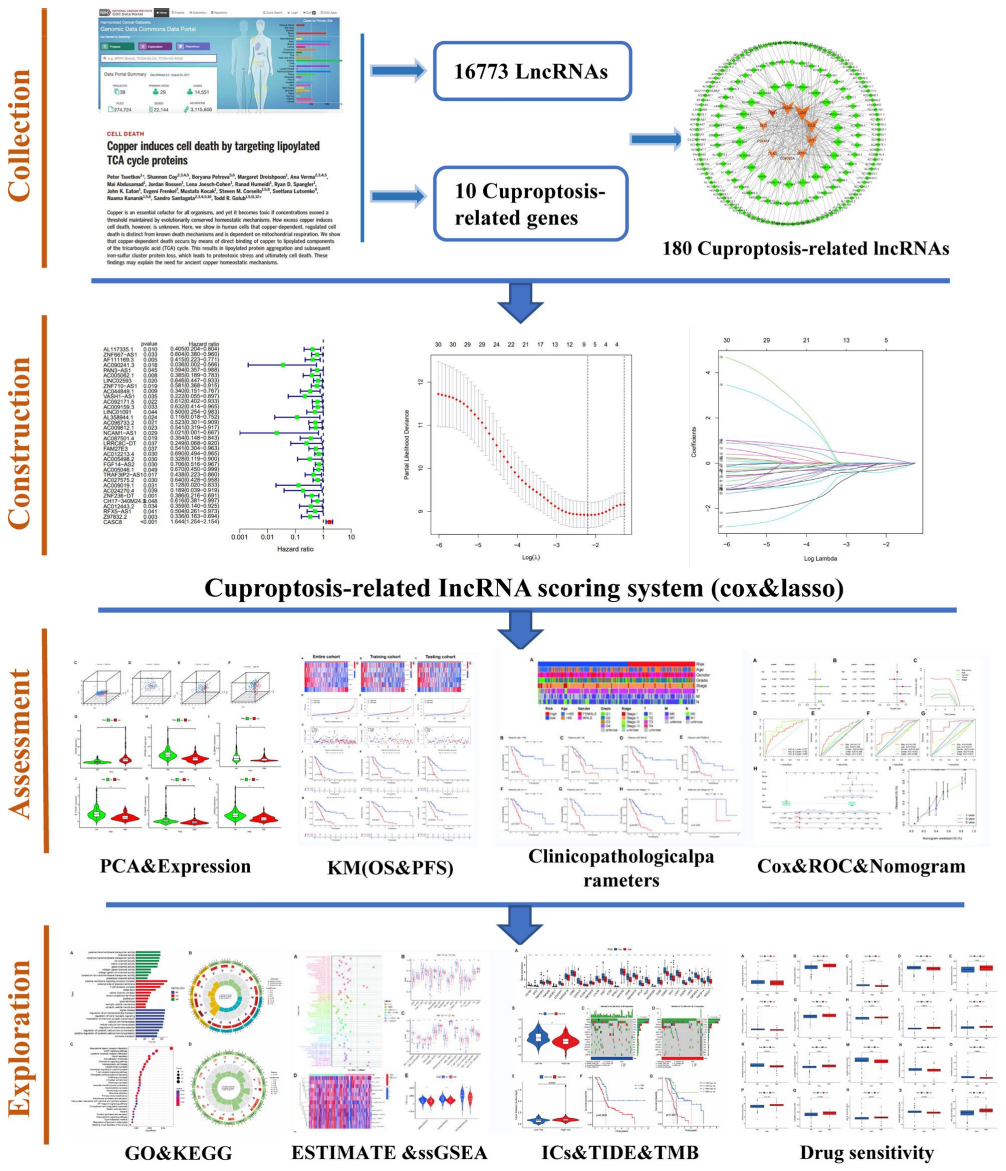
Flowchart of the present research.

**Figure 2:**
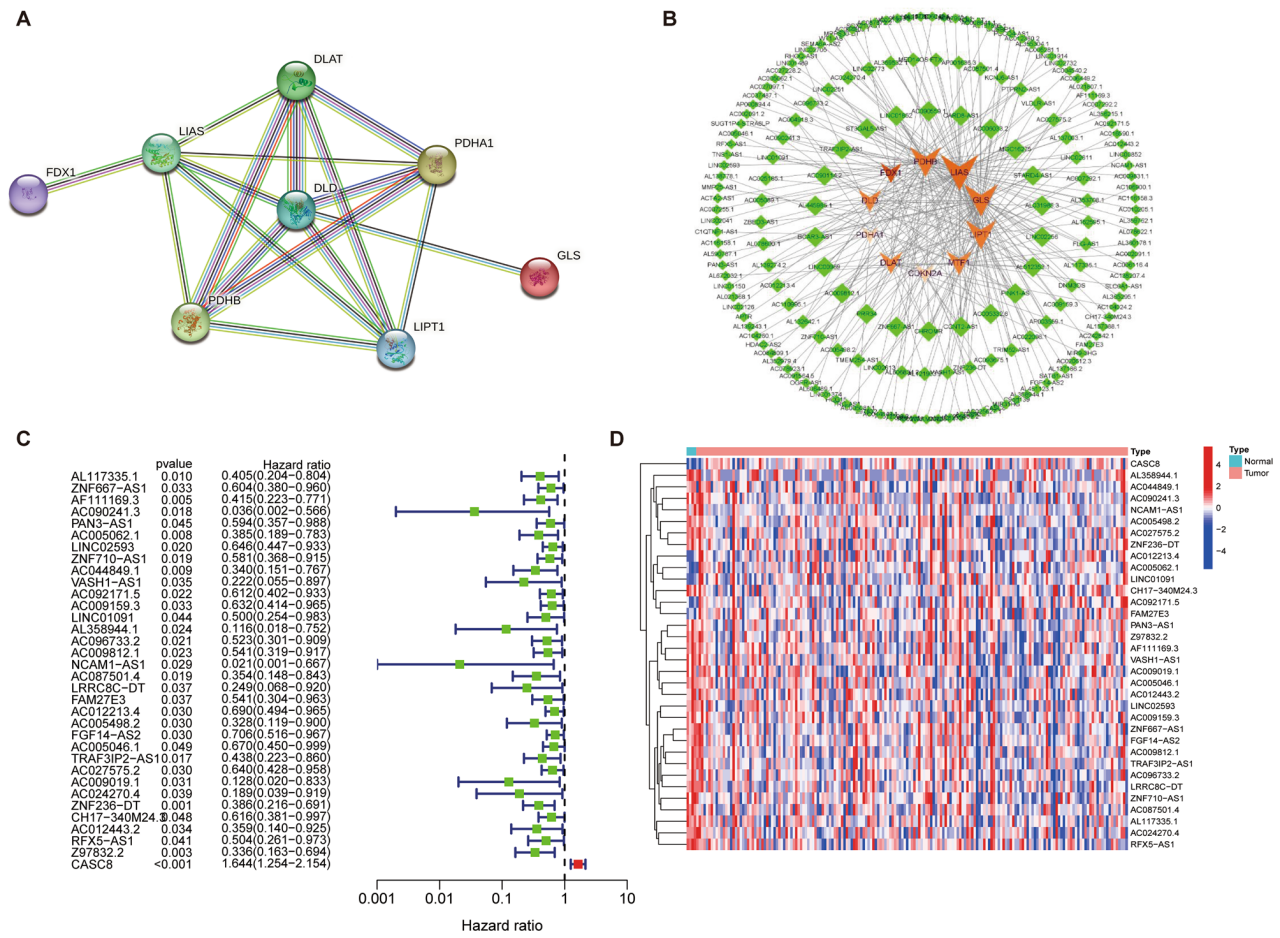
Cuproptosis-related lncRNA in PAAD. (**A)** A PPI network of cuproptosis-related genes. (**B)** Co-expression network of cuproptosis-related genes and lncRNAs. (**C)** Survival analysis forest map of cuproptosis-related lncRNA in the training cohort. (**D)** Heat map of 34 survival-related lncRNAs in the training group at PAAD.

A flow chart is shown in Fig. 1 to directly reflect the ideas and details of this research. The PPI network of cuproptosis-related genes showed that these genes are closely related to various biological processes (Fig. 2A). and then we integrated the TCGA-PAAD transcriptome data and extracted the expression levels of 10 cuproptosis-related genes. A total of 180 cuproptosis-related lncRNAs were obtained by Pearson correlation analysis (Fig. 2B). Next, we randomised the TCGA-PAAD patients into a training and testing cohort in a 1:1 ratio. In the training cohort we screened 34 lncRNAs associated with PAAD survival by Cox survival analysis. We found that except for CASC8 (hazard ratio = 1.644), the other 33 lncRNAs were low-risk lncRNAs [hazard ratio (HR) < 1] (Fig. 2C). Figure 2D shows the expression landscape of 34 survival-related lncRNAs in each TCGA-PAAD sample.

### Construction of the prognostic model

**Figure 3:**
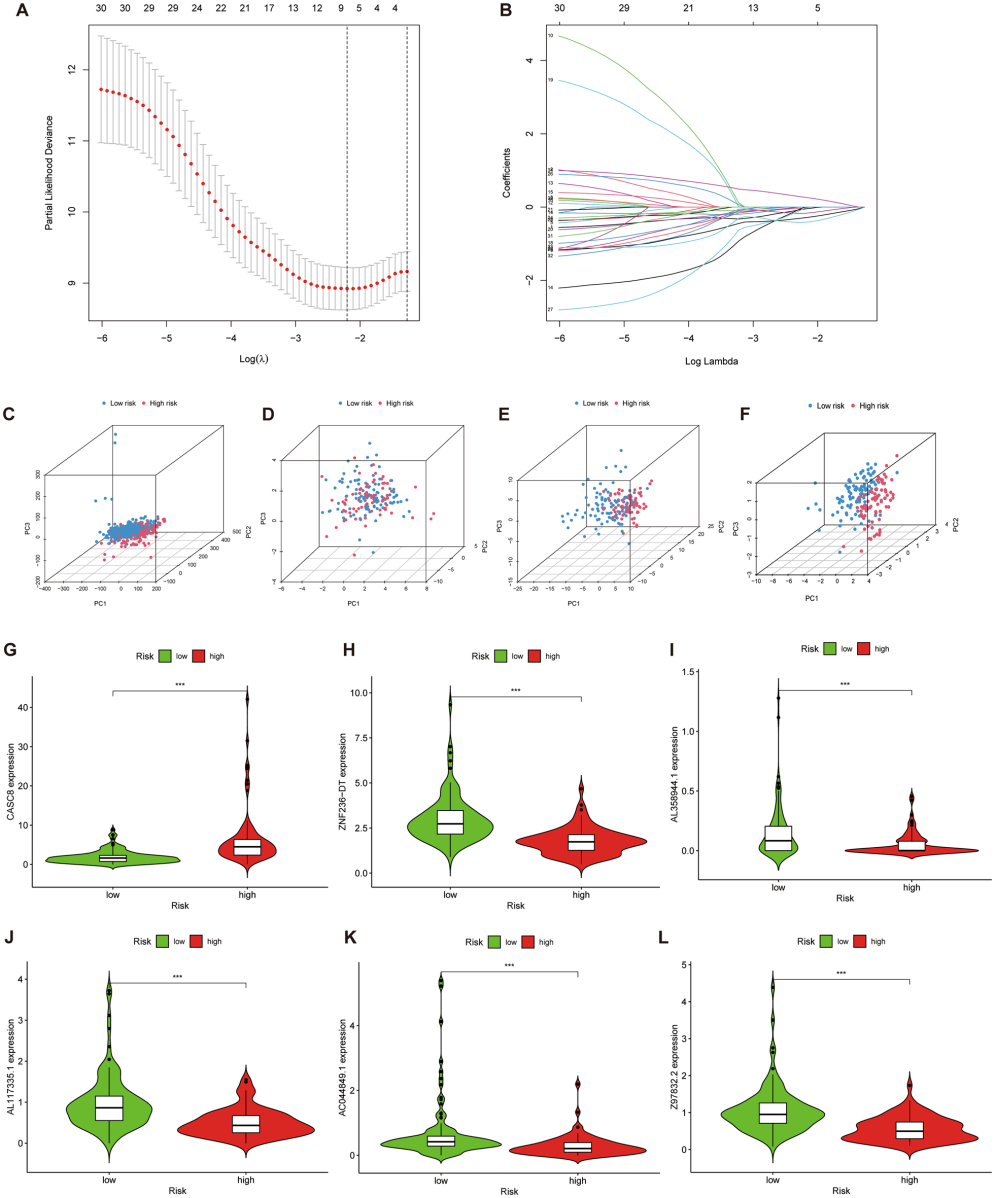
Construction of cuproptosis-related LncRNA scoring system. (**A, B)** LASSO coefficient and partial likelihood deviance of the scoring system. (**C–F**) PCA analysis of allGene, cuproptosis-related gene, cuproptosis-related lncRNA, and risk lncRNA. (**G–L)** Differential expression of six risk lncRNAs in high- and low-risk groups.

Table 1 demonstrates the baseline characteristics of the clinical features (including age, gender, grade, and stage) for the different subgroups. Then, LASSO-COX regression analysis was performed on 34 survival-related lncRNAs (Fig. 3A, B). Cross-validation yielded the minimum λ value and finally mapped six cuproptosis-related lncRNAs (AL117335.1, AC044849.1, AL358944.1, ZNF236-DT, Z97832.2, and CASC8) (Table 2). According to the risk scoring system established above, the prognostic model was constructed. Table 3 shows the association of risk lncRNAs and Curoptosis-Related Genes. The PCA showed that lncRNAs involved in model construction were more obvious than cuproptosis gene, cuproptosis lncRNA, and allGene in the high-low risk group of model differentiation (Fig. 3C–F). Moreover, significant differences were also observed in the expression of six lncRNAs in the high-low risk group (Fig. 3G–L). Given the close association between Copper metabolism and autophagy^36^, we demonstrated the correlation between risk lncRNAs and autophagy-related genes using Sankey plots. (Supplementary Fig. 1).

**Table 1:** Comparison of clinicopathological features between the training and testing cohorts.

Covariates	Type	Total	Testing set	Training set	*p *value
Age	≤ 65	94 (52.81%)	53 (59.55%)	41 (46.07%)	0.0986
	> 65	84 (47.19%)	36 (40.45%)	48 (53.93%)	
Gender	Female	80 (44.94%)	43 (48.31%)	37 (41.57%)	0.4512
	Male	98 (55.06%)	46 (51.69%)	52 (58.43%)	
Grade	G1–2	126 (70.79%)	64 (71.91%)	62 (69.66%)	0.9435
	G3–4	50 (28.09%)	24 (26.96%)	26 (29.21%)	
	Unknown	2 (1.12%)	1 (1.12%)	1 (1.12%)	
Stage	Stage I–II	168 (94.38%)	82 (92.14%)	86 (96.63%)	0.218
	Stage III–IV	7 (3.94%)	6 (6.74%)	1 (1.12%)	
	Unknown	3 (1.69%)	1 (1.12%)	2 (2.25%)	
T	T1–2	31 (17.41%)	16 (17.98%)	15 (15.85%)	0.2036
	T3–4	145 (81.47%)	72 (80.9%)	73 (82.02%)	
	Unknown	2 (1.12%)	1 (1.12%)	1 (1.12%)	
M	M0	80 (44.94%)	34 (38.2%)	46 (51.69%)	0.0818
	M1	4 (2.25%)	4 (4.49%)	0 (0%)	
	Unknown	94 (52.81%)	51 (57.3%)	43 (48.31%)	
N	N0	49 (27.53%)	24 (26.97%)	25 (28.09%)	1
	N1	124 (69.66%)	62 (69.66%)	62 (69.66%)	
	Unknown	5 (2.81%)	3 (3.37%)	2 (2.25%)

**Table 2:** Long noncoding RNA scoring system models associated with cuproptosis.

CRlncRNA	Coef	HR	HR (95%CI)	*p *value
AL117335.1	− 0.5732868	0.405	0.204–0.804	0.009
AC044849.1	− 0.7804579	0.340	0.151–0.766	0.009
AL358944.1	− 1.7008218	0.116	0.017–0.752	0.023
ZNF236-DT	− 0.8142098	0.385	0.215–0.691	0.001
Z97832.2	− 0.7668954	0.336	0.162–0.694	0.003
CASC8	0.5670939	1.643	1.254–2.154	< 0.001

*HR* hazard ratio, *CI* confidence interval.

**Table 3:** Association of lncRNAs and curoptosis-related genes.

CRlncRNA	Curoptosis-related genes	Correlation coefficient
AL117335.1	LIAS	0.319273684
AL117335.1	GLS	0.397339137
AC044849.1	LIAS	0.390888288
AL358944.1	LIAS	0.317588706
ZNF236-DT	LIAS	0.319010685
ZNF236-DT	GLS	0.338474546
Z97832.2	LIAS	0.443451621
CASC8	CDKN2A	0.363102744

### Validation of the model

**Figure 4:**
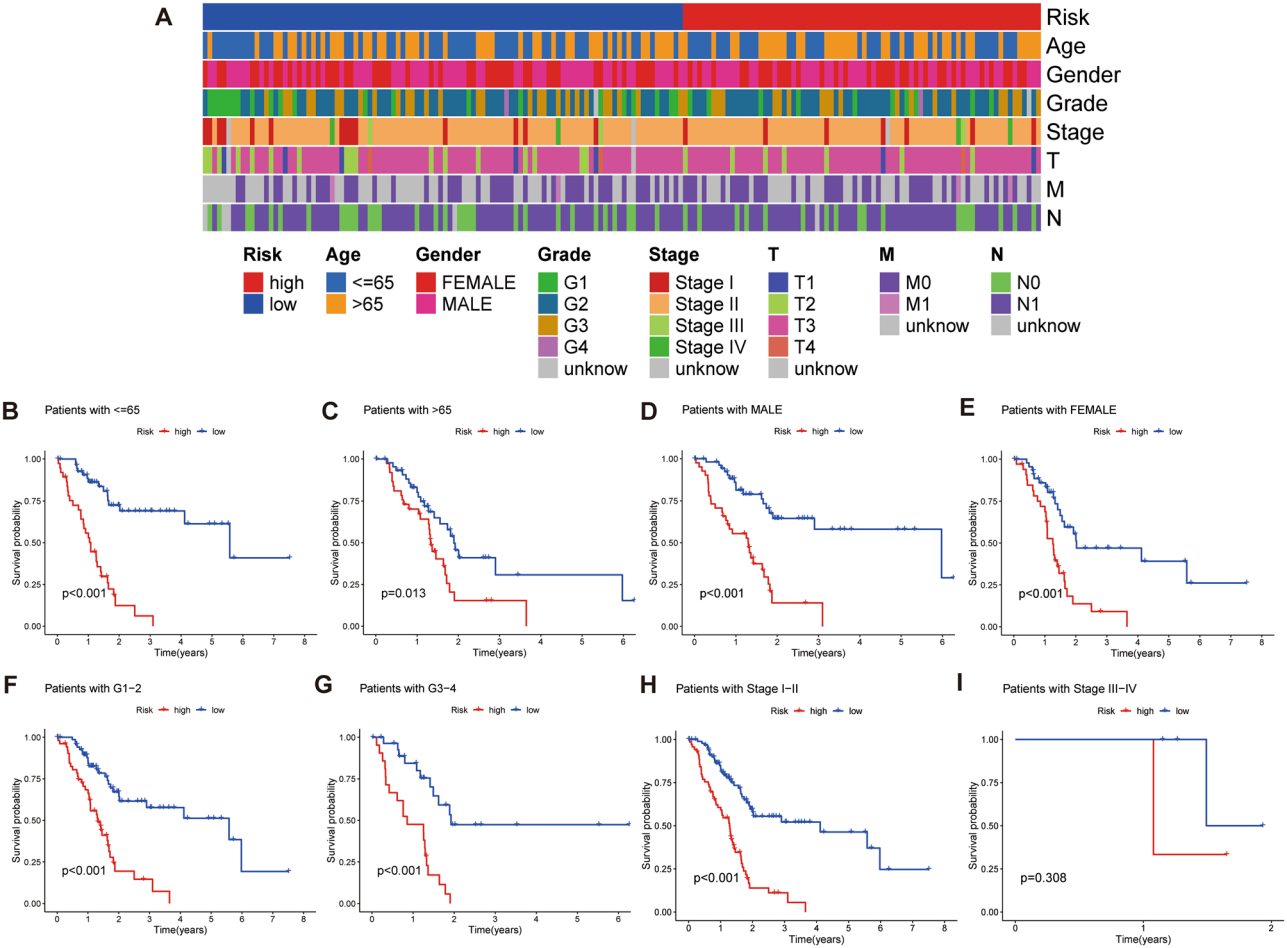
Correlation analysis of clinicopathological parameters in the scoring system. (**A)** Heat map of clinicopathological parameters in the model. (**B, C)** Kaplan–Meier curves for age in high- and low-risk groups. (**D, E)** Kaplan–Meier curves for gender in high- and low-risk groups. (**F, G)** Kaplan–Meier curves for grade in high- and low-risk groups. (**H, I)** Kaplan–Meier curves for TNM stage in high- and low-risk groups.

Figure 4A shows the heat map of different clinical features in the high- and low-risk group. K-M survival analysis of clinical feature subgroups showed that the low-risk group was superior to the high-risk group in age, gender, and grade (Fig. 4B–G). Although the same phenomenon as other clinical features was observed in stages I–II (Fig. 4H), no statistically significant P values were observed in stages III–IV (Fig. 4I). The reason might be the small sample size of stage III–IV cases in the TCGA-PAAD cohort. Nevertheless, we observed a trend toward longer survival in the low-risk group than in the high-risk group. Overall, the model was applicable to PAAD patients with different clinical characteristics.

**Figure 5:**
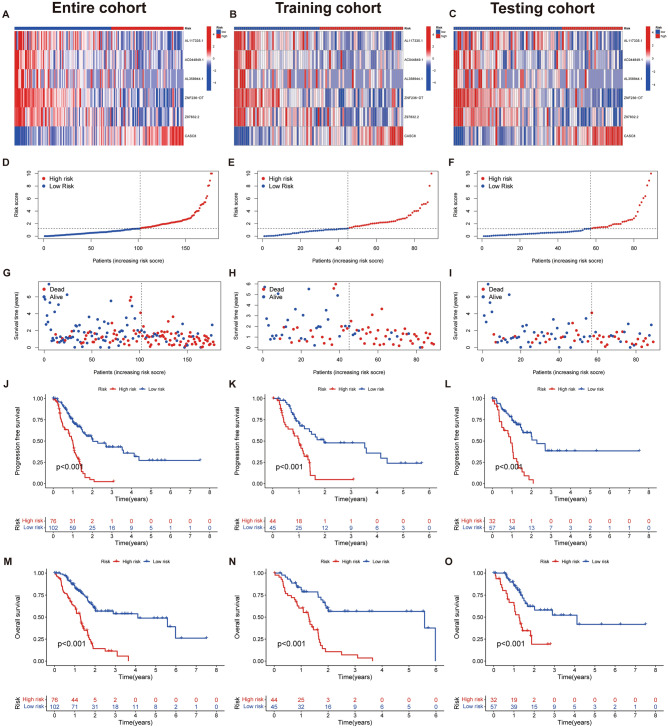
Prognostic values of the cuproptosis-related LncRNA scoring system. (**A–C)** Heat map showing expression levels of the seven lncRNAs in the entire, training, and testing cohorts. (**D–F)** Risk score distribution in the entire, training, and testing cohorts. (**G–I)** Survival time and status in the entire, training, and testing cohorts. (**J–L)** Kaplan–Meier curve for PFS in the entire, training, and testing cohorts. (**M–O)** Kaplan–Meier curve for OS in the entire, training, and testing cohorts.

**Figure 6:**
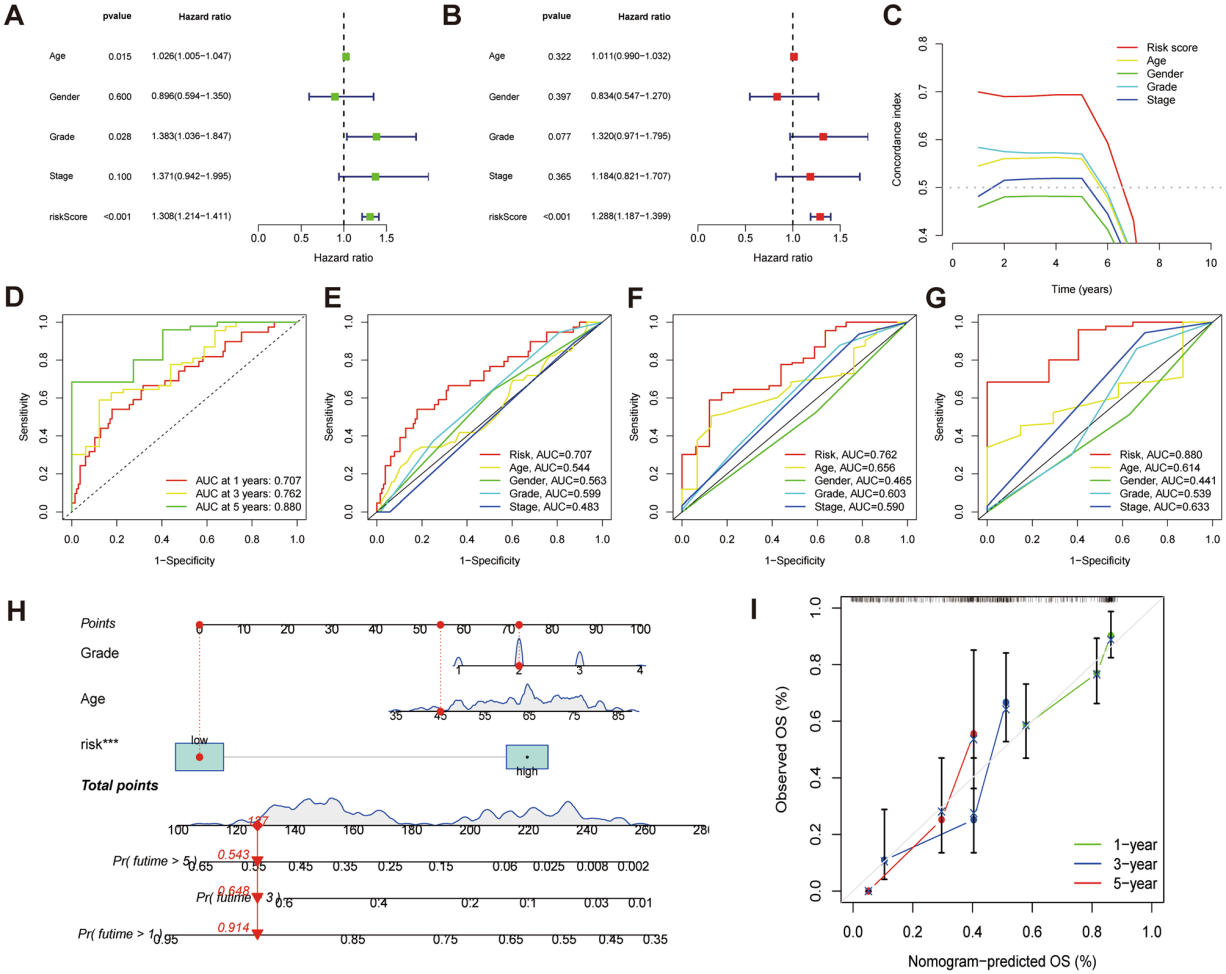
Risk score enrichment pathways and relevance to immune cells. (**A)** Forest plot for univariate Cox analysis. (**B)** Forest plot for multivariate Cox analysis. (**C)** C-index curves of risk score and clinicopathological parameters. (**D)** ROC curves of 1, 3, and 5 years. (**E–G)** ROC curves of risk score and clinical pathology at 1, 3, and 5 years. (**H)** Construction of nomogram for 1-, 3-, and 5-year OS in PAAD patients. (**I)** The calibration curves for 1-, 3-, and 5-year OS.

We assessed heatmaps of expression, risk score, and survival status for the entire cohort, the training cohort, and the testing cohort using the scoring system to further verify the performance of the model. The results showed that the three cohorts were consistent (Fig. 5A–I). Survival analysis showed that the low-risk group had better OS and PFS than the high-risk group (Fig. 5J–O). Age, grade, and risk score were independent factors affecting the prognosis of PAAD patients (Fig. 6A, B). The C-index curve indicated that the risk score was superior to other clinical features in predictive performance (Fig. 6C). In addition, the AUC values at 1, 3, and 5 years were 0.707, 0.762, and 0.880 for the ROC survival curves, respectively (Fig. 6D). Consistent with the C-index curve, the AUC of risk score at 1, 3, and 5 years was also significantly higher than that of other clinical features. Taken together, these results confirmed the reliable clinical predictive accuracy of this model.

### The construction of the nomogram

Based on the results of Cox analysis, we integrated the risk score, clinicopathological parameters (age, grade), and survival data of TCGA-PAAD patients and constructed the nomogram by concretized Cox regression model. The predicted OS of PAAD patients at 1, 3, and 5 years is shown in Fig. 6H. Calibration curves showed that nomogram-predicted values were reliably consistent with actual values.

### Enrichment analysis of DEGs

**Figure 7:**
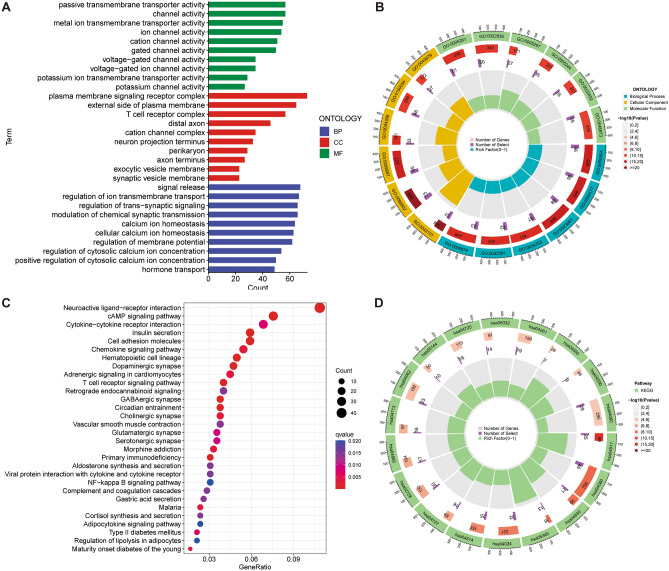
Enrichment analysis of DEGs. (**A, B)** GO enrichment analysis of DEGs. (**C, D)** KEGG enrichment analysis of DEGs.

Through differential gene analysis of the high- and low-risk groups, we finally obtained 1318 DEGs, including 99 upregulated and 1219 down-regulated genes. Then, we explored the biological function of these DEGs by GO and KEGG analyses. GO analysis showed that DEGs were responsible for such activities as T cell activation, T cell receptor complex, metal ion transmembrane transporter activity, and channel activity (Fig. 7A, B). KEGG analysis showed that DEGs were enriched in multiple signaling pathways, such as cytokine-cytokine receptor interaction, cell adhesion molecules, chemokine signaling pathway, and T cell receptor signaling pathway (Fig. 7C, D). In general, DEGs were closely related to ion transport, tumor proliferation and metastasis, and immune regulation.

### Analysis of TIME and prediction of the immunotherapy response

**Figure 8:**
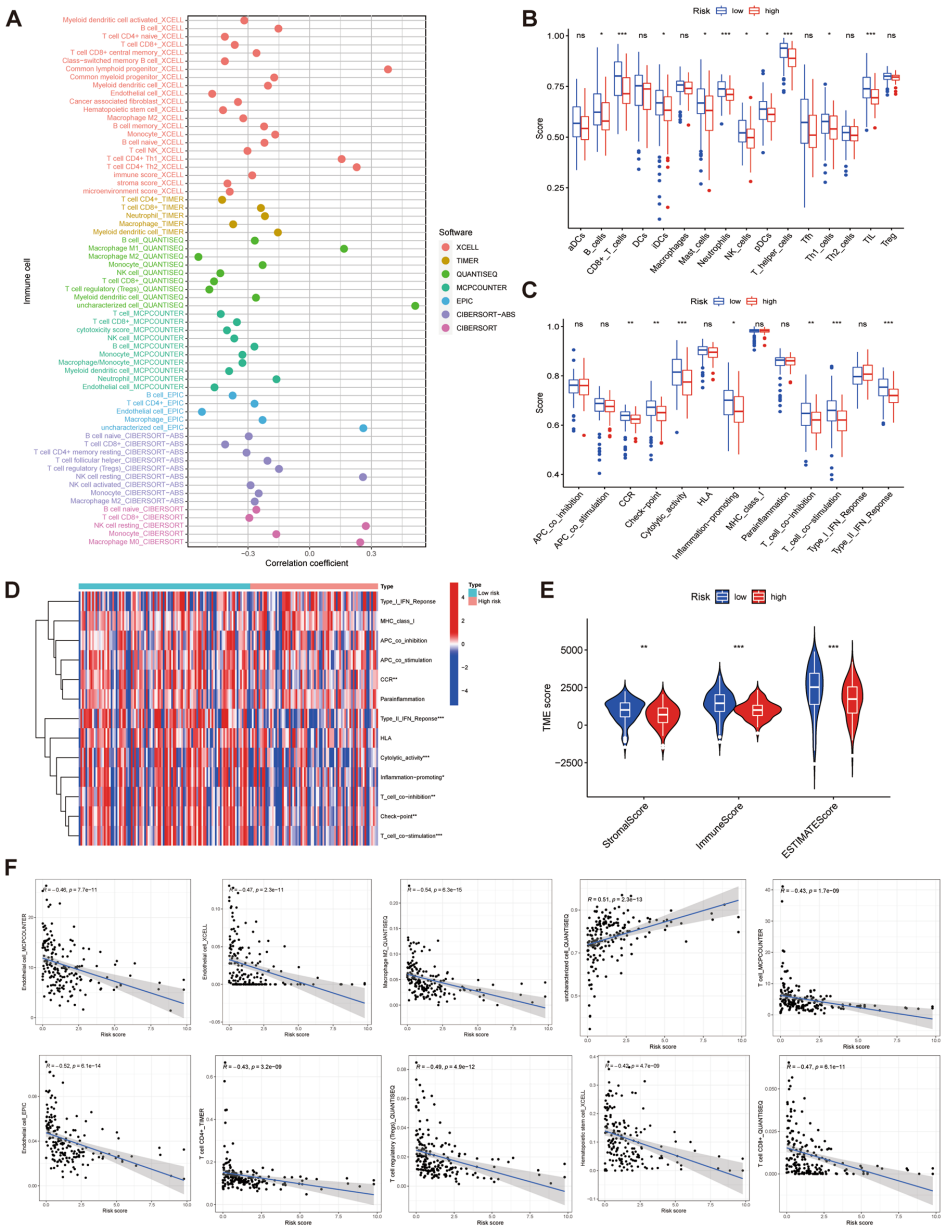
Immunocorrelation analysis of cuproptosis-related lncRNA scoring system. (**A)** Bubble chart of the correlation coefficient between immune cells and risk score. (**B)** Difference analysis of immune cells in high- and low-risk groups. (**C)** Difference analysis of immune function in high- and low-risk groups. (**D)** Heat map of immune function in different risk groups. (**E)** Comparison of tumor microenvironment between high- and low-risk groups. (**F)** Scatter plot of the top 10 absolute values of the correlation coefficients of risk score and immune cells. *P < 0.05, **P < 0.01, and ***P < 0.001.

**Figure 9:**
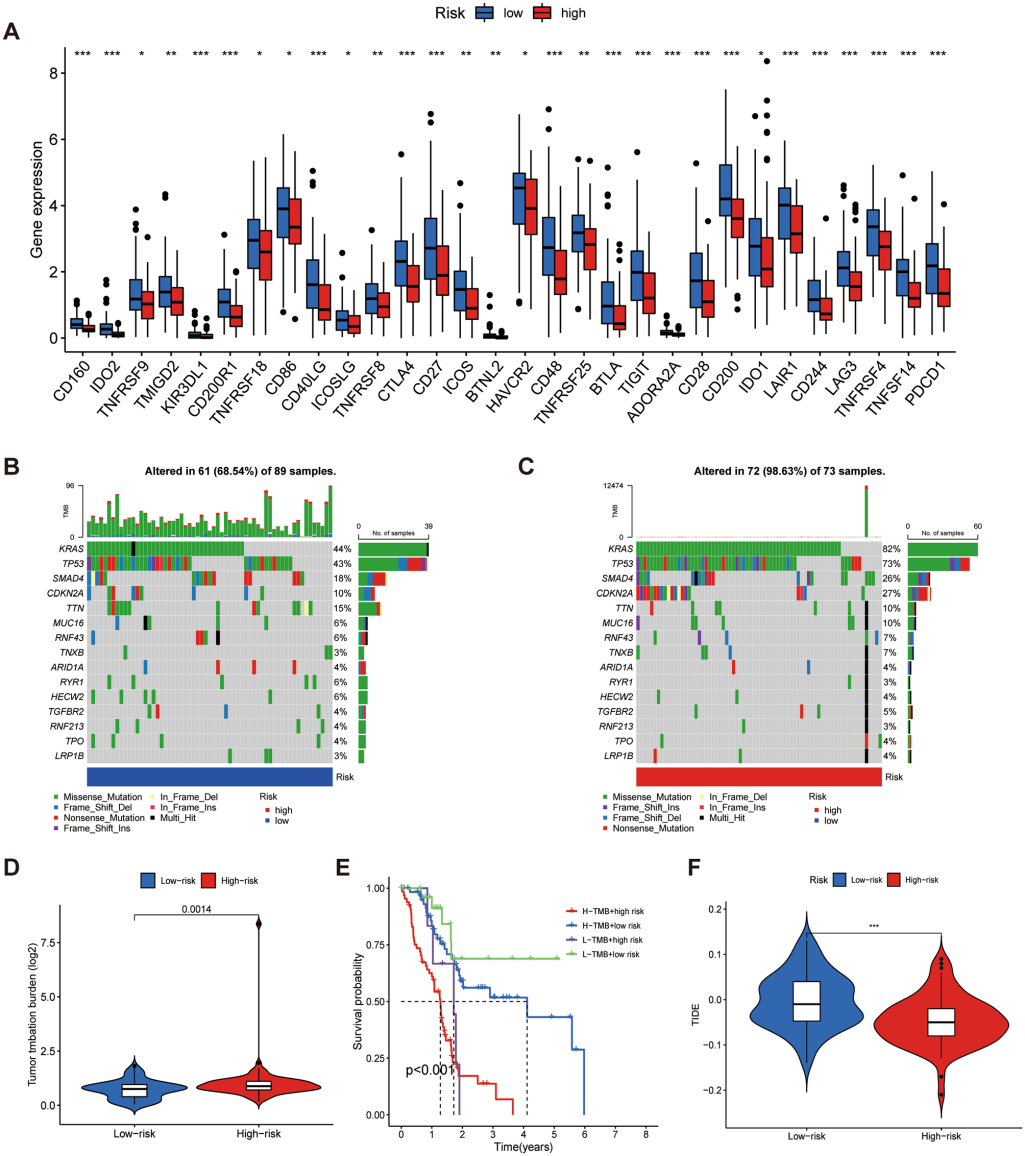
Prediction of immunotherapy response by cuproptosis-related lncRNA scoring system. (**A)** Comparison of immune checkpoint genes in high- and low-risk groups. (**B,C)** Mutation landscape in high- and low-risk groups in PAAD. (**D)** Comparison of TMB expression between high- and low-risk groups. (**E)** Kaplan–Meier survival curves of TMB in high- and low-risk groups. (**F)** Analysis of TIDE.

First, we performed a correlation analysis between risk score and immune cell infiltration abundance. The results showed that a total of 65 immune cells were significantly associated with risk scores (Supplementary Fig. 2). Only a few immune cells were positively correlated with the risk score (Fig. 8A). Then, ssGSEA analysis showed that the content of B cells, CD8^+^ T cells, immature dendritic cells, mast cells, neutrophils, NK cells, plasmacytoid DCs, T helper cells, Th1 cells, and tumor-infiltrating lymphocytes in the low-risk group was higher than that in the high-risk group (Fig. 8B). Immune function analysis also showed that the low-risk group was superior to the high-risk group in CCR (a chemokine receptor), checkpoint, cytolytic activity, promoting inflammation, T cell, T cell costimulation, and type II IFN response (Fig. 8C, D). Based on the ESTIMATE algorithm, the StromalScore and ImmuneScore analysis in different risk groups also obtained consistent results (Fig. 8E). Figure 8F shows a scatter plot of immune cells with the top 10 correlation coefficients. In addition, the expression of 30 immune checkpoint-related genes in the low-risk group was significantly higher than that in the high-risk group (Fig. 9A). The same trend was observed in the TIDE analysis (Fig. 9F). Collectively, these results suggested that patients in the low-risk group had a higher degree of immune cell infiltration and were a potential population to benefit from immunotherapy.

### Mutation landscape of CRLss

There is growing evidence of a close relationship between TMB and immunotherapy response. Therefore, the mutation landscape of PAAD was also under our attention. The waterfall plot showed a lower mutation frequency in the low-risk group (68.54%) than in the high-risk group (98.63%). Additionally, the top three mutated genes in both groups were *KRAS* (H/L: 82%/44%), *TP53* (H/L: 73%/43%), and *SMAD4* (H/L: 26%/18%) (Fig. 9B, C). This is consistent with previous complete exome sequencing of pancreatic cancer^37^. Figure 9D shows that the TMB of the high-risk group was higher than that of the low-risk group. K-M survival analysis showed that patients with low-risk scores and low levels of TMB had better outcomes (Fig. 9E).

### Screening of drugs for potential clinical benefit

**Figure 10:**
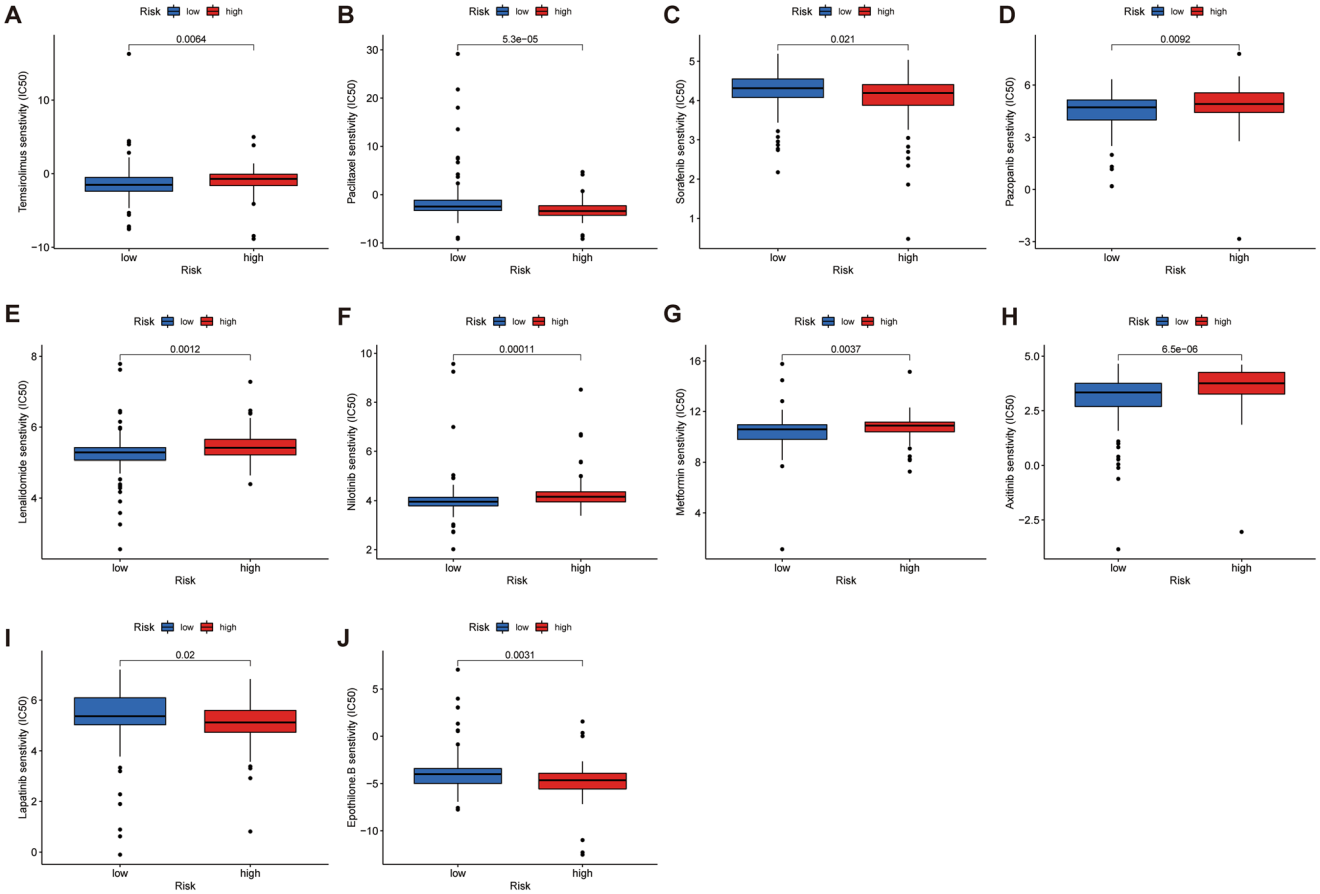
Box plot of drug sensitivity analysis. (**A–J)** Comparison of IC50 values for common clinical drugs in high- and low-risk groups.

We predicted clinical drug response by using the R software package “pRRophetic.” The results showed that there were 56 chemotherapeutic and targeted drugs with differential IC50 values in high- and low-risk groups (Supplementary Fig. 2). We screened clinically common drugs such as chemotherapy and molecular targeting drugs to demonstrate. Compared to low risk groups, We found that low-risk group was better suited to Lenalidomide, Metformin, Nilotinib, Pazopanib, Temsirolimus. While high-risk group was more suitable for Bicalutamide, Epothilone.B, Lapatinib, Paclitaxel, Sorafenib (Fig. 10A–J).

## Discussion

In 2020 global cancer statistics, pancreatic cancer was the 12th most prevalent cancer and the 7th most deadly cancer in the world, and its prevalence and mortality are still increasing every year^38,39^. Predictions indicate that pancreatic cancer will be known as the leading cause of cancer deaths in Europe and the United States by the 2030s and 2040s^39^. The 5-year OS rate is only about 10%, indicating that this is a malignant tumor with a poor prognosis and high invasiveness^40^. In light of the current critical situation, risk stratification and prognosis analysis of PAAD patients with different pathological characteristics by exploring new targets are necessary to improve the level of precision treatment and the efficiency of drug therapy.

Cuproptosis has been identified as a novel form of programmed cell death, which involves the accumulation of copper in cells, impairing mitochondrial metabolic function that leads to cancer cell apoptosis^11^. Several studies have demonstrated the important role of lncRNA in cancer progression and regulation of the TIME^41^. Furthermore, evidence shows that lncRNA can upregulate *STAT1* expression by binding to miRNA, elevating PD-L1 expression in pancreatic cancer. This suggests that lncRNA might be a potential biomarker for immunotherapy in pancreatic cancer^42^. Although the risk-scoring model of lncRNA has been developed to predict the prognosis and TIME state of PAAD^43,44^, the role of cuproptosis-related lncRNA in PAAD remains unclear.

In this study, we obtained six lncRNAs for the construction of the cuproptosis-related lncRNA scoring system and predicted the clinical outcome, immune landscape, and immunotherapy response of PAAD patients. Among them, *CASC8* was significantly correlated with the incidence and survival prognosis of pancreatic cancer. A genome-wide association study (GWAS) of 9,040 patients with pancreatic cancer has identified *CASC8* as a risk factor for the incidence and genetic susceptibility of pancreatic cancer^45^. The competing endogenous RNA mechanism has been constructed using the TCGA-PAAD dataset by Wenjuan Zhu et al., who then identified *CASC8* as a potential therapeutic target for pancreatic cancer^46^. This has also been confirmed by Yingyi Wang et al.^47^. Afterward, the model was systematically validated for its reliable applicability and clinical predictive accuracy in terms of PCA, survival analysis of clinical subgroups and different cohorts, and independent prognostic analysis. Patients in the low-risk group had better clinical prognostic correlation. None of the other five lncRNAs have been reported in PAAD, but given their prognostic value, we believe that their role in pancreatic cancer can be further explored. In addition, we explored the correlation between risk lncRNAs and autophagy genes. Previous studies have shown that copper affects the autophagy process in tumor cells through a variety of pathways, such as the regulation of AMPK-MTOR pathway and the induction of oxidative stress in tumor microenvironment^48,49^. Interestingly, both Cu excess and Cu deficiency affect autophagy^9,50^, so the identification of the role of cuproptosis in autophagy regulation and the identification of key autophagy receptors may be one of the future research directions.

Immune cell infiltration affects the TIME of pancreatic cancer and patient survival^30^. ssGSEA analysis showed that the infiltration of immune cells (such as CD8^+^ T cells, NK cells, T helper 1 cells, and B cells) and immune function (such as promoting inflammation and type II IFN response) in the low-risk group were significantly higher and better, respectively, than those in the high-risk group. CD8^+^ T cells attack cancer cells by recognizing major histocompatibility complex class I-bound cancer antigen complexes^51^. High CD8^+^ T cell infiltration is considered a marker of a “hot” tumor^52^ and a positive factor for immune response and longer survival^30^. NK cells are a type of innate lymphoid cells^53^. It is believed that NK cells induce the differentiation of pancreatic cancer stem cells by secreting IFN-γ and tumor necrosis factor -α, reshaping the tumor microenvironment and inhibiting tumor proliferation^54^. A clinical study has also demonstrated a positive correlation between NK cell infiltration and disease-free survival in resectable pancreatic cancer^55^. As an important member of T helper cell subsets, Th1 cells participate in the activation of M1 macrophages and promote the formation of the inflammatory microenvironment of pancreatic cancer^56^. The role of B cells, as an important component of TIME, is controversial in pancreatic cancer. A study has suggested that differences in the spatial structure of B cells might affect the prognosis of pancreatic cancer patients. Specifically, scattered tumor-infiltrating lymphocytes in the spatial configuration of B cells indicate a worse prognosis compared to tertiary lymphoid tissue (TLT). The mechanism might be related to TLT-type B cells promoting T cell infiltration^57^. However, more research is needed to confirm this finding. Then, we obtained StromalScore and ImmuneScore using the ESTIMATE algorithm. Variance analysis showed consistency with the above-mentioned results. In addition, immune checkpoint genes (including *PDCD1, CTLA4, LAG3*, and *IDO1*) and TIDE analysis also showed an advantage for the low-risk group over the high-risk group. Together, these results suggest that the low-risk group has a better TIME state and could benefit from immunotherapy, tending to represent a “hot” tumor.

TMB is of interest as a novel biomarker for predicting immunotherapy response. At present, it is believed that the high expression of TMB is associated with immune response and good prognosis in some tumors^58^. In 2020, the US Food and Drug Administration (FDA) has approved pembrolizumab for the treatment of TMB-H (TMB ≥ 10 mut/Mb) patients with advanced disease progression and no satisfactory treatment options. However, our study showed that the low-risk group with low expression of TMB had a better immunotherapy response and prognosis. In a study including 36 patients with pancreatic cancer, the immunotherapy response rate has been higher for TMB-L (< 10 mut/Mb) than for TMB-H (≥ 10 mut/Mb)^59^. In contrast, a systematic analysis that included 13 publications has shown a positive correlation between TMB-H and immunotherapy response in pancreatic cancer^60^. Only 1.1% of the included patients achieved the authors’ definition of TMB-H (mean 37.6 mut/Mb), which is much higher than the FDA-defined TMB-H value (≥ 10 mut/Mb). This suggests that the role of TMB as a biomarker of immunotherapy response in pancreatic cancer is limited. However, because of the value of TMB in the prognosis of pancreatic cancer in the CRLss, it is still worth further exploration.

Due to the high heterogeneity of pancreatic cancer^61^ targeted and chemotherapy drugs are now the first-choice treatment in clinical trials. Therefore, we explored the responsiveness to the drugs of different risk groups by constructing a CRLss, which provides a reference for precision treatment. Our study suggests that people in the high-risk group are more suitable for paclitaxel treatment. A phase III randomized clinical study demonstrated that paclitaxel in combination with gemcitabine was effective in improving response rate, OS, and PFS compared to gemcitabine^62^. Despite this, second-line treatment options for pancreatic cancer remain limited and uncertain. Research shows that epidermal growth factor receptor (EGFR) and Human Epidermal Growth Factor Receptor 2 are highly expressed in pancreatic cancer patients^63,64^. Therefore, as a drug that can simultaneously inhibit EGFR and HER-2, lapatinib becomes a feasible choice for second-line treatment of metastatic pancreatic cancer. A phase II clinical study demonstrated a median PFS of 4.0 months and OS of 8.3 months in patients with gemcitabine-refractory pancreatic cancer who benefited from lapatinib in combination with capecitabine, significantly higher than in non-responders. Our study identified the potential beneficiaries of lapatinib through the constructed prognostic model, which provides a basis for clinical medication^65^. Notably, we observed a higher IC50 for metformin in the low-risk group, suggesting that low-risk patients might be more sensitive to metformin. The use of metformin, an old drug for the treatment of diabetes, has been controversial in the treatment of pancreatic cancer^66,67^. In recent years, a growing number of studies have shown the potential value of metformin in pancreatic cancer. For example, metformin modulates the AMPK pathway, downregulates the expression of the fibrogenic cytokine transforming growth factor (TGF)-β, reduces the expression of the pancreatic stellate cell stromal protein α-SMA and collagen, inhibits the production of tumor stroma, and enhances the response to chemotherapy^68^. In addition, it can also inhibit the progression of SMAD4-deficient pancreatic cancer by enhancing AMPK-mediated phosphorylation and ubiquitination degradation of HNF4G protein, providing the possibility of targeted therapy for pancreatic cancer^69^.

Although our study effectively predicted the clinical outcomes and immune landscape of PAAD patients by constructing Cuproptosis-related lncRNA scoring system. But there are still some limitations. Firstly, the validation set in this study was obtained through random grouping within the TCGA database, with a small sample size and some bias. Unfortunately, we did not find an external validation set by searching the established clinical database. Therefore, we expect future clinical studies with a large sample size to demonstrate the applicability of this scoring system. In addition, the lncRNAs screened in this study were obtained by co-expression analysis with cuproptosis-related genes. Therefore, the association of these six lncRNAs with cuproptosis-related genes and their mechanisms of action in PAAD still need to be further confirmed through research.

## Conclusions

In conclusion, the cuproptosis-related lncRNA scoring system had an excellent predictive performance. It could effectively predict the clinical outcome and immune landscape of patients with PAAD. In addition, this study also provides a basis for the selection of chemotherapy, targeted, and ICI drugs, which is essential in the era of precision therapy. However, we still need more clinical trials for further validation.

### Supplementary Information

Supplementary Figures.

## Data Availability

The original contributions presented in the study are included in the article/Supplementary Material. Further inquiries can be directed to the corresponding author.

## Abbreviations

NK: Natural killer
TIME: Tumor immune microenvironment
CRLss: Cuproptosis-related lncRNA scoring system
PAAD: Pancreatic adenocarcinoma
CRGs: Cuproptosis-related genes
TCGA: The Cancer Genome Atlas
PFS: Progression-free survival
PPI: Protein-protein interaction
LASSO: Least Absolute Shrinkage and Selection Operator
AUC: Area under the curve
PCA: Principal component analysis
OS: Overall survival
DEGs: Differentially expressed genes
GO: Gene Ontology
KEGG: Kyoto Encyclopedia of Genes and Genomes
ssGSEA: Single-sample gene set enrichment analysis
TIDE: Tumor Immune Dysfunction and Exclusion
TMB: Tumor mutation burden
IC50: Half-maximal concentration
