# Supplementary material for: Cuproptosis-related lncRNA scoring system to predict the clinical outcome and immune landscape in pancreatic adenocarcinoma
Source: Sci Rep. 2023 Nov 27;13:20870. doi: 10.1038/s41598-023-47223-4 (PMC10682027; doi:10.1038/s41598-023-47223-4)
Supplement: Supplementary file 1 — Supplementary Figures. [file 41598_2023_47223_MOESM1_ESM.doc]

Supplementary Material

## Supplementary Figures

**Supplementary Figure 1** Sankey diagram associated with autophagy-related genes and lncRNA.

**Supplementary Figure 2** Scatter plots of correlation coefficients for different immune cells and risk scores.

**Supplementary Figure 3** Box plots of the correlation coefficients between IC50 and risk scores for different drugs.
